# Representation learning enables robust single cell phenotyping in whole slide liquid biopsy imaging

**DOI:** 10.1038/s41598-025-20514-8

**Published:** 2025-10-21

**Authors:** Amin Naghdloo, Dean Tessone, Rajiv M. Nagaraju, Brian Zhang, Jeffrey Kang, Shouyi Li, Assad Oberai, James B. Hicks, Peter Kuhn

**Affiliations:** 1 Convergent Science Institute in Cancer, University of Southern California, Los Angeles, CA 90089 USA; 2 Department of Aerospace and Mechanical Engineering, University of Southern California, Los Angeles, CA 90089 USA; 3 Department of Biological Sciences, University of Southern California, Los Angeles, CA 90089 USA; 4 USC Norris Comprehensive Cancer, Keck School of Medicine, University of Southern California, Los Angeles, CA 90089 USA; 5 Department of Biomedical Engineering, University of Southern California, Los Angeles, CA 90089 USA; 6 Department of Urology, Keck School of Medicine, University of Southern California, Los Angeles, CA 90089 USA

**Keywords:** Contrastive learning, Single-cell phenotyping, Whole slide imaging, Immunofluorescence microscopy, Liquid biopsy, Cancer imaging, Image processing, Machine learning

## Abstract

Tumor-associated cells in liquid biopsy are promising biomarkers for cancer detection, diagnosis, prognosis, and monitoring. Yet, their rarity, heterogeneity, and plasticity pose challenges for accurate identification and characterization. Enrichment-free whole slide imaging of all circulating cells offers a comprehensive, unbiased approach to capture this phenotypic diversity. However, current analysis methods often rely on engineered features and manual expert review, making them prone to technical variability and subjective bias. To address this, we present a deep contrastive learning framework for feature extraction from whole slide immunofluorescence microscopy images, enabling robust identification and stratification of single circulating cells. Our learned features achieve 92.64% accuracy in classifying diverse cell phenotypes and improve downstream tasks such as outlier detection and clustering. Additionally, our model enables automated identification and enumeration of rare phenotypes, reaching an average F1-score of 0.93 on contrived samples mimicking circulating tumor and endothelial cells, and 0.858 across circulating tumor cell phenotypes in clinical samples. This workflow provides a scalable, reproducible solution for analyzing tumor-associated cellular biomarkers, with strong potential to enhance clinical prognosis and guide personalized treatment strategies.

## Introduction

Liquid Biopsy (LBx) offers a non-invasive method for cancer detection, progression monitoring, and assessment of minimal residual disease by sampling biomarkers from bodily fluids using techniques such as flow cytometry and whole slide imaging (WSI)^1–5^. LBx has advantages compared to traditional tissue biopsy by enabling non-invasive repeated sampling to obtain longitudinal molecular information from tumors^4^. Circulating tumor cells (CTCs), malignant cells disseminated from the tumor into the peripheral bloodstream capable of seeding distant metastatic lesions, are the primary cells studied in LBx^6,7^. Elevated CTC counts in patients are associated with poorer clinical outcomes, including reduced progression-free survival and overall survival, across multiple cancer types^7–11^. CTCs exhibit significant plasticity and heterogeneity and have been categorized into several phenotypes associated with poor patient outcomes, including epithelial-to-mesenchymal transition (EMT)^12,13^, homogeneous and heterogeneous cell clusters^14^, platelet-coated CTCs^15^, and immune-like (CD45+) CTCs^16^. Additionally, previous studies have identified and investigated the utility of other tumor-associated cell populations in peripheral blood^8^, such as circulating endothelial cells^17^, cancer-associated fibroblasts^18^, and macrophages^19^. Recent studies have also demonstrated the prognostic value of immune cell subpopulations in patients with solid tumors^20^. It was shown that white blood cells (WBCs) within heterogeneous CTC clusters may inform tumor–immune interactions and patient stratification^21^, underscoring the value of profiling immune phenotypes in liquid biopsy as well. The study of tumor-associated cell types and phenotypes can provide a more comprehensive view of tumor heterogeneity and evolution in systemic circulation, underscoring the need for approaches that describe their full diversity.

One of the main challenges in cell-based LBx technologies is the extreme rarity of circulating tumor-associated cells, often occurring at rates below one per million WBCs^22^. These cells are typically studied using biophysical enrichment platforms^23^, which enhance sensitivity by targeting predefined phenotypic traits such as cell size or surface marker expression to isolate specific cell populations. Recent developments in deep learning methods have substantially improved the detection of CTCs, facilitating more accurate and rapid identification^24^. While effective for detecting canonical CTCs, this targeted approach reduces the ability to capture the full heterogeneity of the circulating cell population. Alternatively to biophysical enrichment, enrichment-free platforms seek to profile all cells by plating them on slides and using immunofluorescent (IF) markers to differentiate cell phenotypes, generating WSI data that enable comprehensive profiling of the heterogeneous population of tumor-associated cells among all cells^25,26^.

In a typical LBx WSI pipeline, we (i) segment every nucleated cell on the slide, (ii) compute a vector of hand-crafted morphometric and intensity features for each cell, (iii) apply statistical outlier-detection algorithms in this engineered-feature space to flag candidate rare events, (iv) ask an expert reviewer to inspect those flagged objects to identify tumor-associated cells, and (v) determine the phenotypic type of each cell by classification or clustering. Current analytical methods for LBx WSI data rely on engineered features^15,27–29^, yet selecting these features demands substantial apriori knowledge of and depends on the cell phenotypes under study. Engineered features are also sensitive to technical variations common in whole slide imaging, such as blurry regions and inter-instrument variability arising from differences in scanning scope configurations, which can introduce undesirable variance to downstream analyses^30–32^. Moreover, expert-driven annotation and classification are susceptible to subjective bias and lack scalability, limiting throughput and reproducibility of large scale analyses across patient cohorts, as evidenced by only moderate inter-analyst concordance (Cohen’s $\kappa$ of 0.78) when classifying rare circulating cells in enrichment-free IF assays^33^. These limitations highlight the necessity of data-driven workflows to extract robust features from cell images in order to enhance algorithmic methods for identification and phenotypic characterization of tumor-associated rare cells, thereby minimizing human involvement in the process and improving scalability in large scale studies.

Deep representation learning has emerged as an effective approach for learning robust features from image data^34–36^. These models are often trained in a self-supervised manner, allowing them to learn unbiased representations of cell images without the need for extensive labels generated by humans^37,38^. Among representation learning techniques, contrastive learning has been shown to learn powerful discriminative representations across diverse natural images^39^. Several studies in drug discovery have applied contrastive learning to single cell IF microscopy images, where it enables mechanism-of-action classification through single cell representations^40–42^. While these studies demonstrate the utility of self-supervised learning methods for prevalent cell phenotypes, their potential for representing the rare and heterogeneous cell phenotypes present in LBx remains largely underexplored.

In this study, we describe a deep learning framework for extracting robust features from single cell images in LBx that enables resolving the entire phenotypic heterogeneity of the rare tumor-associated cells in WSI data. The approach comprises two main modules: a segmentation model and a feature extraction model. We trained and validated both components using curated well-balanced datasets from patient samples to better represent the rare cell phenotypes. We further demonstrate the utility of the learned features in canonical LBx analysis tasks, including classification of diverse cell phenotypes, outlier detection for identifying rare cell phenotypes, clustering distinct phenotypes, and finally the application of identification and enumeration of rare cell phenotypes in WSI data with severe imbalance using both cell line spiked samples and patient samples. Together, this work provides a framework to learn a robust feature space enabling a highly scalable clinical tool for accurate identification and enumeration of cell biomarkers in LBx and underpinning the unsupervised identification of novel cell phenotypes in WSI data.

## Results

### Overview of the single cell phenotyping framework

**Figure 1:**
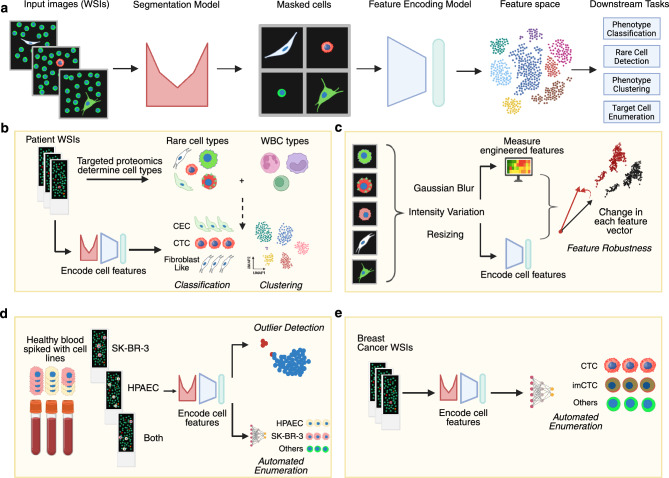
Overview and evaluation of the deep phenotyping framework for liquid biopsy whole slide imaging (WSI) data. (**a**) Schematic overview of the deep phenotyping pipeline and its downstream applications. (**b**) Evaluation of the model’s ability to cluster and classify rare and immune cell phenotypes identified by targeted proteomics. (**c**) Assessment of robustness to common imaging artifacts, including Gaussian blur, pixel size variation, and channel intensity variations. (**d**) Evaluation of outlier detection and automated classification of rare cell types in contrived spike-in samples. (**e**) Application of the framework to classify circulating tumor cell (CTC) phenotypes in breast cancer patient samples. (Created in BioRender. Tessone, D. (2025) https://BioRender.com/6idgfvk)

**Figure 2:**
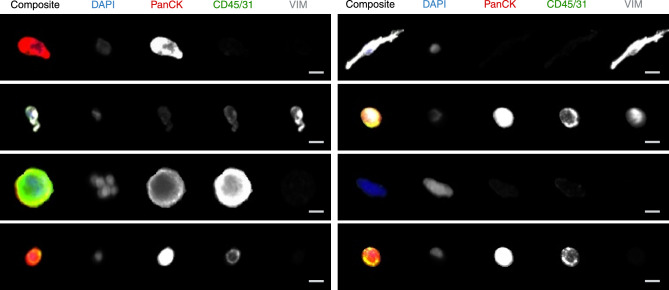
Gallery of diverse cell phenotypes present among rare cells of LBx samples. A scale bar of 10 µm is shown in the bottom right.

Here we establish a deep learning framework designed for robust feature extraction from single cells in WSI data to enhance the performance of common downstream tasks in LBx data analysis (Figure 1a). Our framework leverages two primary trained modules: a segmentation model and a feature extraction model. The breakdown and use of datasets for training and evaluating each module and downstream task (Figure 1b-e) can be found in the Methods section (Table 3) and Supplementary Tables 1 and 2. These WSI data were prepared using a four-color immunofluorescence assay described in detail in the Methods section. Briefly, nucleated cells were plated as a monolayer on glass slides and were stained with DAPI (for DNA), a cocktail of cytokeratins (for epithelial cells), vimentin (for mesenchymal cells and endothelial cells), and CD45 and CD31 (for WBCs and endothelial cells, respectively). Figure 2 represents a gallery of diverse cell phenotypes from the training datasets in this study.

The segmentation model, based on a U-Net architecture provided by Cellpose^43^, was trained on the training split of Dataset A (Table 3) and evaluated on the test set. The model showed improved accuracy in detecting individual cells compared to the general-purpose Cellpose model. We evaluated performance using the F1-score across different thresholds of intersection-over-union (IoU), a metric that measures how well predicted cell masks overlap with their ground truth masks. The trained model achieved higher F1-scores for IoU values between 0.5 and 0.8, indicating better agreement in object-level segmentation, and showed comparable performance at other IoU thresholds (Supplementary Figure 1). Further details can be found in the Methods section. The feature extraction module was then trained using segmented and sampled rare cells and WBCs from WSI data of 25 patients (Datasets B and C, Table 3, Supplementary Figure 2). WBCs were controllably depleted with a pretrained binary classifier that achieved 99.37% accuracy, thereby creating a balanced dataset for training the feature encoding model (Dataset B, Table 3). We evaluated the quality and performance of learned features in a number of downstream tasks, including classification of known single cell phenotypes, rare cell detection for novel phenotype discovery, clustering to characterize unknown cell phenotypes, and enumeration of known tumor-associated cells in WSI data both in cell line-derived experimental samples and patient samples (Figure 1b-e). The details of model architectures, their training data and implementations are described in the Methods section.

### Linear classification confirms discriminative capacity of learned features on a broad range of cell phenotypes

Deconvolving the heterogeneous phenotypes of rare tumor-associated cells as well as immune cells is essential for scalable clinical analysis and for associating distinct cell populations with clinical outcomes, leading to biomarker discovery in preclinical settings. To assess how well the learned feature space captures phenotypic variation relevant to these tasks, we first perform supervised classification, commonly referred to as linear probing, as a standard evaluation in representation learning. This approach measures the ability of the learned representation to organize known phenotypes in a way that supports accurate downstream classification using labeled data. In order to perform this task, we assembled a ground truth dataset comprising a heterogeneous population of cell phenotypes, verified by a single cell targeted proteomics analysis through imaging mass cytometry (IMC) and augmented with phenotypically matched cells collected from additional samples (Dataset D, Table 3). This dataset comprises both rare tumor-associated cells and diverse WBC subpopulations, annotated using additional proteomic markers and distinct morphological features.

The ground truth dataset covered seven distinct rare cell phenotypes; canonical epithelial CTCs^44^, immune-like CTCs (imCTCs)^16^ , platelet-coated CTCs (pcCTCs)^15^, circulating endothelial cells (CECs)^44^, megakaryocyte-like (Mega-like) and fibroblast-like (Fibro-like) cells, and morphologically abnormal nuclei with no other IF marker expression (L-Nuclei). The megakaryocyte-like and fibroblast-like cells are cell groups that exhibit IMC marker profiles consistent with megakaryocytes and fibroblasts, respectively. We further expanded the ground truth dataset of cell phenotypes by adding cells from three major immune cell subclasses including lymphocytes (Lymph), monocytes/macrophages (Mono), and granulocytes (Gran). Table 1 represents all cell phenotypes in immunofluorescent images along with their additional proteomic markers used to describe their phenotype.

**Table 1:** Description of cell phenotypes in ground truth dataset.

Cell phenotype	Immunofluorescent markers	Additional IMC markers
CTC	DAPI+ CK+	CK8/CK18+
imCTC	DAPI+ CK+ CD45/31+	CK8/CK18+ CD45+ CD31-
pcCTC	DAPI+ CK+ CD45/31+	CK8/CK18+ CD45- CD31+
CEC	DAPI+ VIM+ CD45/31+	CD45- CD31+
Megakaryocyte	DAPI+ CD45/31+	CD31+ CD61+ CD68+
Fibroblast	DAPI+VIM+	CD31- CD34+ CD44+ Fibronectin+
Large Nucleus	DAPI+	N/A
Lymphocyte	DAPI+ CD45/31+	CD3+ or CD20+ or CD56+
Monocyte/Macrophage	DAPI+ CD45/31+	CD3- CD20- CD56- (CD14+ or CD68+)
Granulocyte	DAPI+ CD45/31+	CD3- CD20- CD56- CD14- CD68-

**Figure 3:**
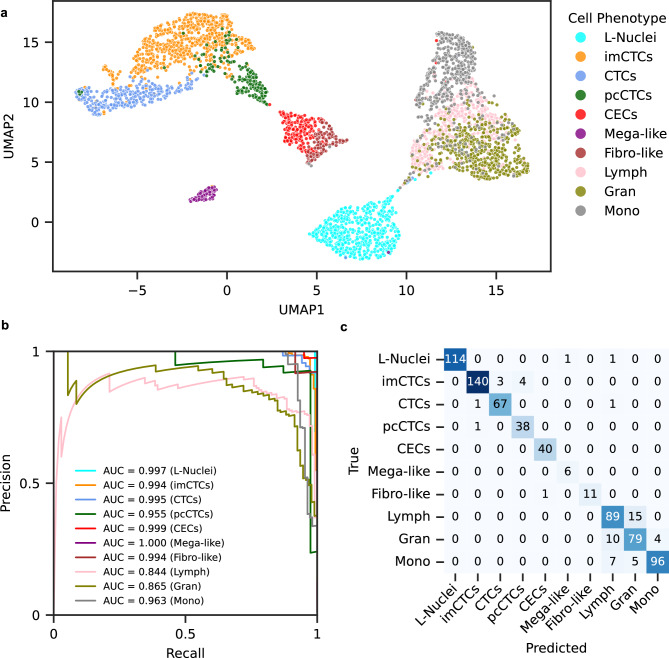
Learned feature representations stratify diverse single cell phenotypes. A ground truth dataset of diverse cell phenotypes was assembled to evaluate the performance of features provided by the deep phenotyping framework. (**a**) UMAP projection of cells, colored by ground truth cell type annotations. (**b**) Precision-recall curves for each cell type on the held-out test set, with areas under the curve (AUCs) indicated in the legend. (**c**) Confusion matrix of the logistic regression multi-class classifier on the test data.

We used our framework to collect learned feature representations of this dataset. A two-dimensional UMAP projection of these cell features is presented in Figure 3a. We then trained a logistic regression classifier on 80% of the dataset to predict cell phenotypes. When tested on the remaining 20% of the dataset, the model achieved an accuracy of 92.64% in classifying cell phenotypes. The micro-average area under the precision-recall curve was 0.969 and the macro-average was 0.961 (Figure 3b). We further assessed the area under the receiver-operating characteristic (ROC) curve, which achieved a micro-average of 0.996 and a macro-average of 0.994. These results are comparable to those obtained with engineered features (Supplementary Figure 3), reflecting on the high classification fidelity of learned features.

Among all rare cell phenotypes, the lowest area under the precision-recall curve is 0.955 (pcCTCs), whereas all other rare event classes have areas under the curves greater than 0.99. In addition, all classes have areas under the ROC curve of greater than 0.98. Further, we noted that the majority of misclassifications within the rare event classes naturally occur between cell phenotypes with subtle differences in their IF images (Figure 3c). Among the 13 misclassified rare events, 7 imCTCs are misclassified as either canonical epithelial CTCs (3) or pcCTCs (4). Both pcCTCs and imCTCs are epithelial cells and share identical IF identification criteria (Table 1), making them exceedingly challenging for humans to distinguish in the absence of proteomic data.

### Contrastive learning features are robust to technical variations

Technical variations in WSI, including differences in optical focus, pixel size, and channel-specific intensity, can introduce non-biological variation that degrades the consistency of single-cell features. To test whether the learned feature space is robust to such variations, we applied a series of controlled perturbations to the test images (Dataset D, Table 3) that simulate common scanner-related batch effects: Gaussian blur to mimic regional blur as a result of out-of-focus imaging, per-channel intensity variation to account for staining or detector variability, and slight spatial resizing to reflect differences in pixel size across imaging systems.

**Figure 4:**
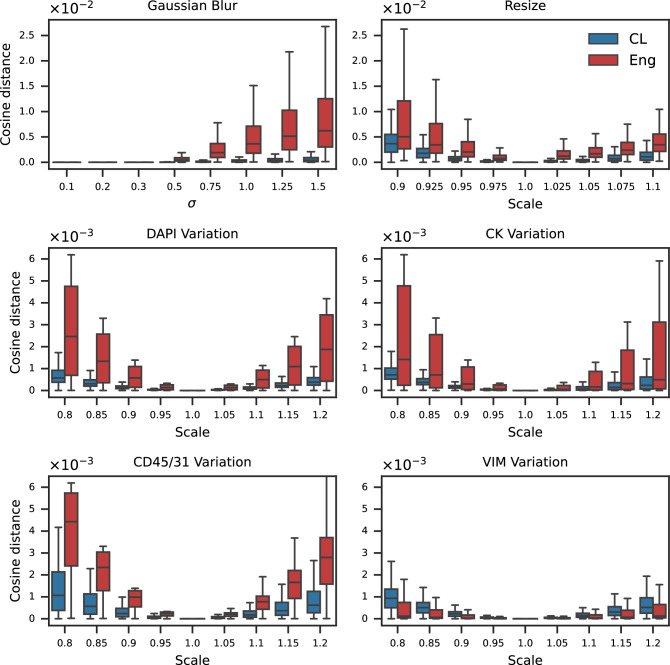
Learned features are generally more robust than engineered features to scanner-mimicking image perturbations. Cosine distances between original and perturbed single-cell features were computed for both engineered (Eng) and learned (CL) feature spaces across three perturbation types: Gaussian blur, image resizing, and single-channel intensity variation.

For each perturbation, we computed the cosine distance between the feature vectors of original and perturbed images using both engineered and learned features. The results, shown in Figure 4, demonstrate that learned features consistently exhibit lower sensitivity to perturbations than engineered features, indicating greater robustness to technical variation. This trend held across all image channels and perturbation types, with one minor exception in intensity variation for VIM channel where the learned features showed slightly higher drift compared to engineered features. This reduced robustness is likely due to the relatively low representation of VIM+ cells in the training set.

### Learned features enhance outlier detection of rare tumor-associated cell phenotypes in WSI

Outlier detection is a common downstream application in enrichment-free LBx to overcome the rarity limitation of tumor-associated cells primarily in peripheral blood while preserving the cellular heterogeneity. However, the effectiveness of this approach varies across rare cell phenotypes due to differences in how these cells are represented in the feature space relative to common WBCs. Unlike classification, which targets known phenotypes with labeled data, outlier detection enables discovery of unforeseen or unannotated cell types in complex, unlabeled samples, an important capability in real-world liquid biopsy settings. Here, we assess the performance of our learned features in detecting outliers and compare their performance to engineered features.

As a benchmark dataset to evaluate the effect of feature sets in outlier detection, we generated contrived WSI data by spiking SK-BR-3 or HPAEC or both cells into healthy blood samples at concentrations of 1:10,000 WBCs (0.01%) (Dataset E, Table 3). SKBR3 is a human breast cancer cell line that mimics canonical epithelial CTCs, whereas HPAEC is a human pulmonary artery endothelial cell line that mimics tumor microenvironment CECs. All slides were stained and imaged with the same protocol as described in Methods, generating 9 WSI data. This dataset was processed via our trained segmentation module and manually annotated as SK-BR-3, HPAEC, or other based on marker expression. We tested both learned features extracted from our framework and engineered features to compare their effect on the performance of the outlier detection task.

**Figure 5:**
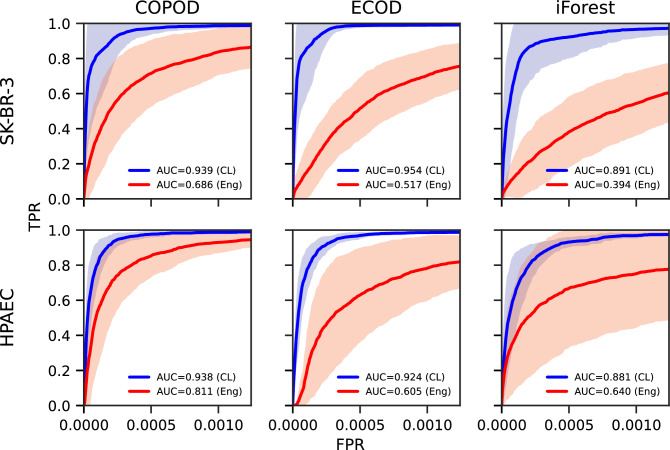
Learned feature representations enhance identification of rare cell phenotypes via outlier detection. Three outlier detection methods (COPOD, ECOD, iForest) were applied to identify rare tumor-associated cells using either learned (CL) or engineered (Eng) feature representations. The relative frequency of target cells among detected outliers (top 2500 outliers) is shown for each method and feature space.

We evaluated three distinct outlier detection methods on the feature sets: Copula-Based Outlier Detection (COPOD)^45^, Empirical-Cumulative-distribution-based Outlier Detection (ECOD)^46^, and isolation forest (iForest)^47,48^. In each test, we assessed the number of identified SK-BR-3 and HPAEC cells among the top 2,500 outliers from a total of about 2.5 million cells (~0.1% of all cells) identified in each WSI data (Figure 5). We observed that across all three outlier detection methods and two phenotypes, the learned feature space resulted in a higher relative frequency of target cells among the identified rare events compared to engineered features. This trend was consistent across methods, indicating reproducibility of performance gains from learned features. For SK-BR-3 cells, ECOD yielded the highest area under the mean ROC curve of 0.954 utilizing the learned features, as compared to 0.517 for the engineered features. HPAEC cells were best detected utilizing COPOD with an area under the mean ROC curve of 0.938, as opposed to 0.811 for engineered features. Additionally, the detection rates across the two cell types, SK-BR-3 and HPAEC, were comparable when using the learned features. In contrast, the engineered feature space exhibited uneven detection performance, with substantially better recovery of HPAEC cells than SK-BR-3 cells.

### Learned features improve clustering performance in the presence of data imbalance

While rare cell detection methods are employed to address the challenge of extreme imbalance between immune and tumor-associated cells, the identified rare cells can still be imbalanced across phenotypes. This data imbalance can hinder downstream analyses, particularly unsupervised clustering as a common technique when investigating novel rare cell phenotypes for biomarker discovery. Clustering methods are often sensitive to data imbalance that may exist among rare cell phenotypes. While development of advanced clustering algorithms aims to address this limitation to some extent, the characteristics of the feature space on which the clustering is performed can help improve the impact of imbalance on clustering results. Here, we assess the performance of our learned features in clustering under imbalanced conditions compared to engineered features.

**Figure 6:**
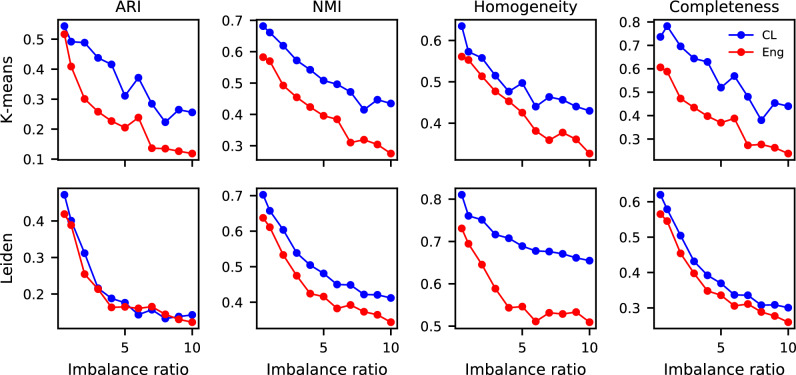
Learned features improve clustering performance on imbalanced cell phenotype data. Clustering performance of learned (CL) and engineered (Eng) feature representations was evaluated using two unsupervised algorithms, K-means and Leiden community detection, on a ground truth dataset of cell phenotypes. Experiments were conducted across a range of data imbalance ratios, defined as the ratio of immune cells to rare cells, from 0.5 to 10. Clustering quality was assessed using adjusted Rand index (ARI), normalized mutual information (NMI), homogeneity, and completeness scores.

Leveraging the ground truth dataset of cell phenotypes (Dataset D), we evaluated the performance of two commonly used clustering methods, K-means and Leiden community detection, in delineating distinct cell phenotypes using learned features and engineered features. We defined the data imbalance ratio as the ratio of total immune cells to total rare cells and performed the experiment across a range of ratios from 0.5 to 10. We quantified the performance of each clustering result by calculating standard metrics of adjusted-rand index (ARI), normalized mutual information (NMI), homogeneity, and completeness (Figure 6). The description of evaluation metrics and hyperparameters of the clustering algorithms can be found in the Methods section.

Across all imbalance ratios, learned features demonstrated improved clustering performance over engineered features. For example, with K-means clustering at the lowest imbalance ratio, learned features (CL) achieved completeness value of 0.74 compared to 0.61 for engineered features. Similar trends were observed for NMI (0.68 versus 0.58), homogeneity (0.63 versus 0.56), and ARI (0.54 versus 0.52). As the imbalance ratio increased, performance declined across all methods; however, learned features maintained a performance margin of 5%–25% over engineered features, particularly in homogeneity and NMI metrics. Aside from similar performance regarding ARI score, this advantage persisted with Leiden clustering, where learned features yielded a 8%-14% improvement in all other metrics at lowest ratios and retained higher performance across increasing imbalance ratio.

### Learned features enable accurate enumeration of rare cell phenotypes in WSI

Having established the utility of learned features compared to traditional features, we next sought to evaluate their performance in a common downstream application. Accurate enumeration of rare tumor-associated cell phenotypes is a critical application of cell-based LBx, particularly for clinical decision-making and biomarker assessment. However, reliable identification remains challenging due to the extreme rarity of these cells and potential presence of technical artifacts and non-tumor rare events in WSI data. We evaluated the efficiency of our learned feature representations in detecting distinct rare cell phenotypes in two different experiments: (1) spike-in experiments with contrived samples of known cell lines at defined rarity, and (2) patient samples containing two different CTC phenotypes, canonical epithelial phenotype (CTCs) as well as immune-like CTCs (imCTCs).

**Figure 7:**
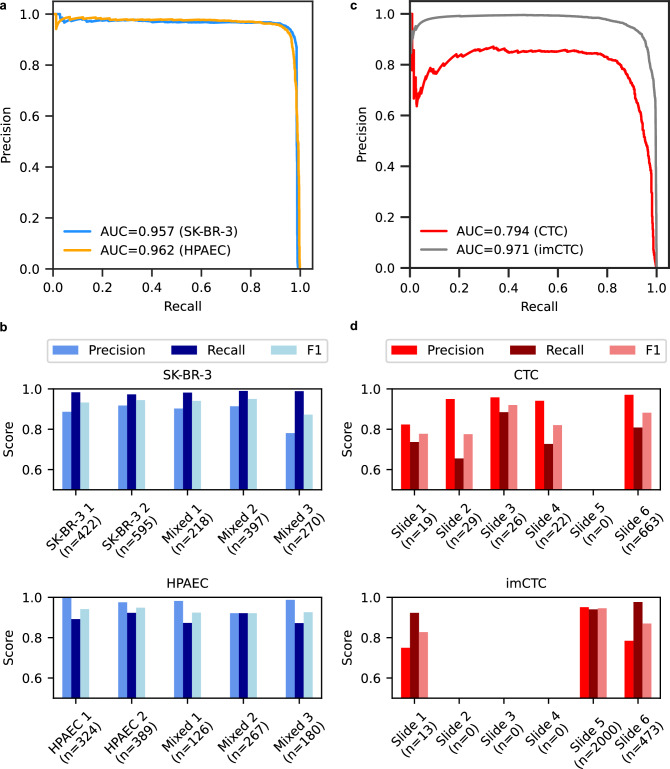
Learned features support accurate enumeration of rare cell phenotypes in both contrived and patient-derived WSI data. (**a**) Precision-recall curves for classification of SK-BR-3 and HPAEC cell lines in spike-in WSI data. (**b**) Per-slide performance metrics (precision, recall, F1-score) for SK-BR-3 and HPAEC classification across test WSIs containing different spike-in configurations. (**c**) Precision-recall curves for detection of CTC and immune-like CTC (imCTC) phenotypes in breast cancer patient WSIs. (**d**) Per-slide performance metrics for CTC and imCTC classification across six held-out patient WSIs.

In the first setting, two contrived WSIs containing either SK-BR-3 or HPAEC cells were used to train a multilayer perceptron classifier on learned features to classify each single cell as SK-BR-3, HPAEC, or other (Dataset E). We evaluated this model on 7 additional WSIs composed of various combinations of these cell lines and controls, totaling over 15 million single-cell events, including 1,902 SK-BR-3 cells and 1,286 HPAEC cells. The classifier achieved high overall precision (0.886 for SK-BR-3, 0.971 for HPAEC) and recall (0.982 for SK-BR-3, 0.903 for HPAEC), with an area under the precision-recall curve (AUPRC) of 0.957 and 0.962 for SK-BR-3 and HPAEC, respectively (Figure 7a). Further, classification performance remained consistently high across different slides, with an average F1-score of greater than 0.93 on both cell lines (Figure 7b).

To assess its potential clinical utility, we applied the framework to a cohort of 8 samples from late-stage breast cancer patients with manually annotated CTCs and imCTCs (Dataset F). A classifier was trained on learned features extracted from two samples, and tested on six held-out samples comprising over 13 million cells. Despite significantly variable CTC frequencies across samples, from 19 to 663 CTCs and from 13 to 2000 imCTCs (representing 0.006% and 0.018% of total events), the model achieved overall precision values of 0.965 for CTCs and 0.912 for imCTCs, with recall values of 0.801 and 0.947, respectively. The AUPRCs were 0.794 for CTCs and 0.971 for imCTCs (Figure 7c). Slide-level analysis showed consistent performance, with average F1-score of 0.835 for CTCs and 0.881 for imCTCs (Figure 7d).

## Discussion

Cell-based liquid biopsies hold a great promise for longitudinal monitoring and minimal residual disease identification in cancers. A variety of enrichment-based and enrichment-free approaches have revealed substantial heterogeneity and plasticity among rare, tumor-associated cells in circulation^13–15,17–19^. Deconvoluting the heterogeneity of these circulating tumor-associated cells is feasible in enrichment-free platforms and can yield critical patient-specific insights and drive the discovery of novel biomarkers, ultimately improving patient outcomes^15^. Currently, analysis of distinct cell phenotypes is conducted through classical image processing methods using engineered cell image features, which are sensitive to technical variations, and often necessitate additional human annotation, which is prone to significant human bias.

Here, we developed a deep learning framework that leverages contrastive learning to develop features of single-cell LBx images to discriminate cellular populations in a robust and precise manner from unenriched LBx samples. Our results show that learned features are capable of accurately separating diverse tumor-associated phenotypes as well as immune cell subclasses. While previous work demonstrated comparable performance in supervised classification of 6 different cell types in EpCAM-enriched samples^49^, their enrichment-based approach limited phenotypic diversity of single cells. By training and applying our feature encoding model on enrichment-free WSI data, we overcome this limitation and demonstrate strong performance in classification of 10 distinct classes, including 3 different CTC phenotypes. This enables the systematic study of multiple distinct tumor-associated phenotypes in parallel, along with distinct immune cell subpopulations that may be tumor-reactive, immunosuppressive, or otherwise indicative of tumor-associated signaling within the peripheral blood microenvironment. Such granularity is essential for capturing the full heterogeneity of circulating cells in liquid biopsy.

Our results show that contrastive learning produces feature representations that are more robust than engineered features to a range of scanner-related image perturbations, including blur^50,51^, intensity variation, and slight resizing. This robustness enhances the reliability of downstream single-cell phenotyping tasks by reducing sensitivity to technical variability and is especially valuable in rare cell studies, where suboptimal image quality can significantly impact downstream analyses, including phenotyping classification, clustering, and outlier detection. The observed exception in the vimentin channel underscores the importance of sufficiently balanced channel-specific signal distribution in training data representation. Overall, these findings support the use of contrastive learning for generating phenotypically meaningful and technically resilient features in cell-based imaging.

Further, we demonstrate the capability of the learned features in outlier detection, a common task in the study of rare cells. The learned features yielded a higher concentration of rare cell lines among detected outliers in our contrived samples than engineered features. The consistently improved performance across multiple outlier detection methods suggests that the observed gains are attributable to the learned feature space rather than the specific detection algorithm. Moreover, the comparable detection performance of SK-BR-3 and HPAEC cells in the learned feature space contrasts with the biased detection favoring HPAECs when using engineered features. This indicates that the learned features promote more phenotype-agnostic performance, enabling balanced detection of rare cells across diverse phenotypes. Engineered feature space can, in principle, be selected for improved detection of each rare cell phenotype as well. However, this process requires prior knowledge of the target phenotypes, hindering the study of previously unknown rare cell phenotypes.

Moreover, learned features show significant improvements in the capacity to cluster cell phenotypes over engineered features in the presence of data imbalance, a natural property of cell phenotype populations in WSI data. Given the sensitivity of clustering algorithms to class imbalance and the ongoing development of specialized methods to address this limitation, our framework offers a robust feature space, as an orthogonal improvement, that enhances the ability to identify and discover novel cell phenotypes using current algorithms.

After establishing the utility of learned features in comparison to engineered features, our results revealed that the learned features enable precise identification and enumeration of two different target cell lines, mimicking CTCs (SK-BR-3) and CECs (HPAEC). Our work on enrichment-free samples resulted in F1-scores of 0.934 and 0.937 for SK-BR-3 and HPAEC, respectively, achieving equivalent performance to size-based enrichment studies that leverage supervised classification directly on cell images in the enriched cell population^52^. These results demonstrate that size-based microfiltration might be excessive for robust identification of circulating rare cell phenotypes. Moreover, relatively consistent performance across independent samples in our results demonstrate that the features we provide enable reproducible classification and enumeration of target cell phenotypes, offering reliable application of features in enumeration-based studies of cell biomarkers in patient cohorts.

Finally, we extended our classification analysis to patient samples by demonstrating that the learned feature representations enabled accurate identification and enumeration of two phenotypically distinct circulating tumor cell CTC populations. Although the model was trained on data from only two patients, it achieved average F1-scores of 0.835 and 0.881 for CTCs and imCTCs, respectively, when evaluated on independent patient samples. This strong performance, despite limited training data, highlights the capacity of feature space to generalize to unforeseen patient samples. A recently published work proposes a supervised model which has been trained on EpCAM-enriched patient samples, achieving an F1-score of 0.886^53^ with a limited detection capacity to only canonical epithelial CTCs. While having comparable performance scores, our framework analyzes enrichment-free WSI data and allows for the identification of a broader spectrum of multiple tumor-associated cell phenotypes. Together with its high performance, these results highlight the potential of our features for a broad clinical applicability. To the best of our knowledge, this is the first application of deep learning-derived features for simultaneous enumeration of multiple CTC phenotypes from patient data. The F1-scores we report suggest that representation-learned features can reduce subjectivity in rare-cell identification. In a related enrichment-free liquid-biopsy study we measured expert inter-rater reliability at Cohen’s $\kappa$ of 0.78^33^, with other enrichment-based inter-analyst reliability studies finding comparable task difficulty (median $\kappa$ of 0.83)^54^. While further validation across larger and more diverse cohorts is warranted, these findings suggest a promising potential for scaling this framework in support of rare cell phenotype enumeration and classification in translational studies.

One of the limitations of this work is that the feature encoding model was trained on a small number of patient samples, limiting the generalizability of the existing feature space. Inclusion of a larger, more diverse patient population across multiple cancer types and stages in training, will improve the performance of our general-purpose LBx feature extraction. Further, the limited training data of only two patient samples for CTC enumeration hinders the performance of classification models across novel patients samples. Given the inter- and intra-patients variability of CTCs and heterogeneity of cells other than CTCs^55^, including more samples in the training data can significantly improve the accuracy of classification models in identifying the target cells.

Taken together, our findings demonstrate that contrastive learning yields single-cell IF image representations capable of facilitating robust phenotype classification, outlier detection, cell clustering, and supervised enumeration of circulating tumor-associated cells, even under realistic imaging artifacts and severe class imbalance. By removing the dependency on experimental enrichment and manual curation, this framework enables high-throughput pipelines for circulating rare-cell analysis that are more scalable, reproducible, and efficient than current practice. The same feature space can also be aggregated in the future with methodologies such as multiple-instance learning to learn patient-level biomarkers, paralleling recent advances in histopathology^56,57^. Overall, these results position deep phenotyping of single-cell, enrichment-free, liquid-biopsy images as a powerful, generalizable approach for cancer monitoring and biomarker discovery.

## Methods

### Patient cohorts

Patient samples included in this study were assembled from multiple, previously published independent cohorts spanning several IRB-approved clinical studies. A collection of WSI data were selected from de-identified patient samples to provide a diverse population of rare events for training the segmentation and feature extraction modules as well as to cover the heterogeneity in immune populations for training the immune cell classifier used for depleting WBCs to curate the training data for the feature extraction module. All samples were collected under protocols approved by the respective institutional review boards (IRBs), and written informed consent was obtained from all participants in accordance with the Declaration of Helsinki. Prior to analysis, all samples and associated metadata were de-identified to ensure patient privacy. Cohort-specific details, including cancer type/condition, study reference, number of samples used in this study, and IRB protocol number are summarized in Table 2. The studies listed in Table 2 were conducted in accordance with the Declaration of Helsinki and were approved by the Institutional Review Board at the University of Southern California, as detailed for each individual study.

**Table 2:** Overview of patient cohorts and study IRBs.

Cohort	Condition	# Slides	IRB Protocol # (approval date)	Reference
1	Breast cancer (all stages)	150	UP-14-00523 (10/21/14)	Shishido, et.al^58^.
			HS-16-00435 (02/01/19)	
2	Bladder cancer	57	HS-19-00886 (01/08/20)	Shishido, et.al^59^.
			UP-14-00592 (12/01/14)	
			HS-15-00434 (07/20/15)	
3	Metastatic prostate cancer	43	UP-16-00691 (12/21/16)	Chai, et.al^15^.
4	Normal blood donor	38	UP-14-00592 (12/01/14)	Setayesh, et.al^60^.
5	Non-small cell lung cancer	33	HS-17-00854 (07/31/19)	Bai, et.al^61^.
6	Colorectal cancer	31	UP-14-00592 (12/01/14)	Narayan, et.al^62^.
7	Upper tract urothelial carcinoma	30	HS-19-00886 (01/08/20)	Shishido, et.al^63^.
8	COVID-19	14	HS-21-00556 (12/07/21)	Qi, et.al^64^.
9	Oligometastatic breast cancer	6	UP-14-00592 (12/01/14)	Woodward, et.al^65^.
10	Metastatic breast cancer	3	UP-17-00882 (03/28/24)	Higa, et.al^66^.
11	Multiple myeloma	1	UP-19-00333 (08/07/19)	Setayesh, et.al^67^.

### Sample collection and preparation

LBx samples, predominantly peripheral blood, (~8 mL) were collected in 10 mL tubes (Streck, Cell-free DNA BCT) at the clinical site and processed within 48 hours of collection^58^. Upon receiving the samples, cell counts were measured using hematology analyzer (Medonic) to determine the volume of blood needed for each slide to plate about 3 million cells per slide. In processing, isotonic ammonium chloride (A649-3, Fisher Scientific) erythrocyte lysis was conducted on each sample and the remaining cellular fraction is was plated on custom-made cell adhesive glass slides (Marienfeld, Lauda, Germany), generating between 8 and 16 slides per blood specimen, depending on the cellular density of the sample. Cells were plated on each slide, incubated with 7% BSA, dried, and stored at -80$^\circ$ C for future studies^58,68,69^.

### Immunofluorescence staining protocol

Slides were thawed for one hour and stained using the IntelliPATH FLX autostainer (Biocare Medical LLC) using a 4-color staining protocol. Each slide was first fixed with 2% PFA (stock 16%, 15710, Electron Microscopy Sciences) in 1x TBS (TWB945, Biocare Medical LLC) for 20 minutes. Then, the slides were incubated for 4 hours with 2.5 $\mu$g/mL of a mouse IgG1 anti-human CD31 monoclonal antibody conjugated to AlexaFluor® 647 (clone: WM59, MCA1738A647, BioRad) and 100 $\mu$g/mL of goat anti-mouse IgG monoclonal Fab fragments (115–007–003, Jackson ImmunoResearch). Cells were then permeabilized for 5 minutes using 100% methanol (A412-1, Fisher Scientific) and blocked for 20 minutes with 10% filtered goat serum (S26-LITER, Emd Millipore) in TBS to prevent non-specific interaction of primary and secondary antibodies. Goat serum was used as an antibody dilution buffer to the following steps of the staining protocol. Slides were incubated for 2 hours at room temperature with a primary antibody cocktail including mouse IgG1/IgG2a anti-human pan-cytokeratin monoclonal antibodies (CK1, CK4, CK5, CK6, CK8, CK10, CK13, CK18) (C2562-.5ML, Sigma-Aldrich), and CK19 antibody (M088801-2, Dako North American), rabbit IgG anti-human vimentin monoclonal antibody conjugated to AlexaFluor® 488 (VIM, 9854BC, Cell Signaling Technology), and mouse IgG2a anti-human CD45 monoclonal antibody conjugated to AlexaFluor® 647 (CD45, MCA87A647, AbD Serotec). Finally, slides were incubated with goat anti-mouse IgG1 AlexaFluor® 555 (A21127, Thermo Fisher) secondary antibody and 4 ’6 diamidino-2-phenylindole (DAPI, D1306, Thermo Fisher), mounted in a glycerol-based mounting media (BP229-1, H5123, ICN10274750, Fischer Scientific), and cover-slipped (12545J, Fisher Scientific)^15^.

### Image acquisition

Stained slides were then imaged using an in-house automated fluorescence scanning microscope with 10x objective and 100x overall magnification, yielding 2304 frames per slide for each of the four immunofluorescence channels^68^. Collected images were stored as 16-bit TIFF format (1362x1004). The total image size for one slide is approximately 16 GBs.

### Engineered feature extraction

The engineered features for all analyses were extracted using the EBImage package (version 4.48) in R (version 4.4.2) provided the image and the cell mask. The 368 features included cell size, eccentricity, haralick texture features and intensity statistics including mean, median, standard deviation and different quantiles from each individual channel image as well as combination of channel pairs.

### Imaging mass cytometry

Slides that were stained with IF markers were restained with a multiplexed panel of metal-conjugated antibodies to characterize diverse cell phenotypes. The imaging mass cytometry (IMC) analysis was conducted on a CyTOF Helios imaging mass cytometer (Fluidigm). A table of antibody clones used in these studies and their associated cell type can be found in Supplementary Table 3. Antibodies not directly conjugated to metals were conjugated in the lab with a Maxpar antibody labeling kit. Single cells were segmented through a trained ilastik (version 1.3.3) model and ion count per cell was extracted using CellProfiler (version 3.15) in a pipeline developed by the Bodenmiller lab^74^. Thresholds for each marker were defined by observing the ion count histograms.

### Datasets

For this study, we collect six unique datasets for training and testing distinct elements of liquid biopsy analysis. Table 3 summarizes their provenance, size, class balance, train/test splits, and the specific analyses in which each appears. The datasets described here are subsets derived from 406 whole slide images drawn from 277 previously published patient samples.

**Table 3:** Summary of the six datasets employed in this study.

Code	Dataset	Composition	Purpose	Split (train:test)
A	Segmentation	787 image-mask pairs	Developing segmentation model	80%:20%
B	WBC Depletion	18,298 total cells:	Developing WBC depletion model	80%:20%
		9,149 WBC		
		9,149 non-WBC		
C	Representation Learning	25 slides:	Developing feature encoder model	80%:20%
		117,281 rare cell		
		11,250 WBC		
D	Ground-Truth Phenotypes	23,225 total cells:	Phenotype classification	80%:20%
		2,166 rare cell	Robustness analysis	
		30,059 WBC	Clustering analysis	
E	Spiked-In WSI	9 slides:	Outlier detection analysis	2 slides:7 slides
		2,641 SK-BR-3	Imbalanced phenotype identification	
		1,691 HPAEC		
		22.5 M other		
F	CTC Patient Cohort	8 slides:	Phenotype identification in patients	2 slides:6 slides
		5,220 imCTC		
		936 CTC		
		13 M other		

Codes A-F are referenced throughout the Results to trace data provenance for every experiment.

#### Dataset A: Segmentation

This dataset (Table 3) was assembled to retrain and evaluate the Cellpose segmentation model. The image crops of size 256 $\times$ 256 were selected such that they contained at least 1 rare cell. For each crop, we utilized the pretrained Cellpose model, cyto3, to generate an initial instance mask and then corrected each mask manually using the Cellpose graphical user interface (version 3.0.11).

#### Dataset B: WBC Depletion

Whole slides images derived from liquid biopsy samples contain a super majority of WBCs. To prevent the downstream representation-learning stage from being dominated by WBCs, we collected single cell images (Table 3) across published datasets in breast cancer^58^, prostate cancer^15^, multiple myeloma^70^, bladder cancer^59^, colorectal cancer^62^, non-small cell lung cancer^72^, upper tract urothelial carcinoma^63^ and non-cancerous post-acute covid syndrome^64^ to train a WBC depletion model.

#### Dataset C: Representation Learning

Twenty-five enrichment-free whole-slide images (23 peripheral blood, 2 bone marrow aspirates) were passed through the segmentation model and WBC depletion model to yield 117,281 segmented non-WBCs (Table 3). We note that in addition to true rare cells, the process collects significant technical noise, including blurry cells and out-of-distribution staining noise. From each slide, an additional 450 WBCs were randomly subsampled to generate a total dataset of 129,071 single-cell images, stored as 75×75×5 crops (Four IF channels + mask), with the cell centered in the crop.

#### Dataset D: Ground Truth Phenotypes

To evaluate the quality of the feature space extracted by the SimCLR encoder, manually identified tumor-associated rare cells and additional WBCs (Table 3) were characterized by IMC to constitute a gold-standard phenotype benchmark^13,16,44^. This dataset was further expanded by adding phenotypically matched cells collected by trained analysts. Cells were characterized into a total of 10 phenotypes including epithelial CTCs, immune-like CTCs, platelet-coated CTCs, endothelial cells, megakaryocytes, fibroblasts, large nuclei, lymphocytes, monocytes, and granulocytes based on a set of protein markers (Table 1). The entire dataset was later used for clustering analysis as well.

#### Dataset E: Spiked-In WSIs

Nine WSIs were generated by spiking SK-BR-3 and/or HPAEC cells at 1:10,000 into healthy donor blood (3 SK-BR-3 only, 3 HPAEC only, 3 both, Table 3). SK-BR-3 cells and HPAEC cells were manually annotated from each WSI data using annotateEZ, an annotation tool developed in-house (see code availability section). SK-BR-3 cells were selected for their large size, expression of cytokeratin, and negativity for vimentin and CD45/CD31. HPAEC cells were selected for their expression of CD45/CD31 (multiplexed in one channel), vimentin, and large size. We have previously shown that HPAEC cells have variable expression levels of cytokeratin^44^.

#### Dataset F: CTC Patient Cohort

Eight WSIs derived from late-stage breast cancer patients were collected to test the quality of the feature representations under genuine clinical conditions (Table 3). CTCs and imCTCs were manually annotated from each WSI data using annotateEZ. CTCs were selected for their large size, expression of cytokeratin, and negativity for vimentin and CD45/CD31. imCTCs were selected for their expression of cytokeratin, CD45/CD31 (multiplexed in one channel), and large size.

### Segmentation model

We trained a custom segmentation model using the Cellpose backbone architecture, with no pretrained weights^43^ on the training subset of Dataset A. To evaluate instance segmentation performance, we computed the object-level F1-score at multiple intersection-over-union (IoU) thresholds^71^. This metric quantifies the accuracy of predicted object masks by comparing them to ground truth annotations based on their spatial overlap. The IoU is defined as the ratio between the area of intersection and the area of union of a predicted and a ground truth object mask. A predicted object is considered a true positive (TP) if its IoU with a ground truth object exceeds a specified threshold, and each ground truth object can be matched to at most one prediction. Unmatched predictions are counted as false positives (FP), and unmatched ground truth objects as false negatives (FN). For each IoU threshold, F1-score is calculated by Equation 1. This evaluation captures both the detection and localization accuracy of instance-level predictions, with increasing IoU thresholds enforcing stricter spatial agreement for a match.

1 $$\begin{aligned} F_1 = \frac{2 \times \textrm{TP}}{2 \times \textrm{TP} + \textrm{FP} + \textrm{FN}} \end{aligned}$$

### WBC depletion classifier architecture and training

The WBC depletion classifier was trained using a PyTorch implementation of a CNN. The model consisted of four batch-normalized convolutional layers, activated by ReLU, each followed by a Max-Pooling layer of size (2,2), and a 2-layer dense network to reduce the features to class logits. The cross-entropy loss function was used to train the model. A learning rate of 0.0001 was used with the Adam optimizer to optimize the training. The model was trained for 25 epochs on one RTX4090 GPU, monitoring train and validation loss curves on training subset of Dataset B. AUROC and PR-ROC curves were generated on the test subset.

### Feature encoding model

The architecture of the representation learning model comprises a CNN encoder architecture and a two-layer projection network. The CNN contains four convolutional layers, each followed by batch normalization. After the last convolutional layer a Max-Pooling Layer with a 2×2 kernel, with stride of 2 is applied, followed by an Adaptive Pooling Layer with a 1×1 kernel. ReLU is used as the activation function following all convolutions. The features are then flattened before being passed to the two-layer projection network, where the loss is calculated and back-propagated.

For every minibatch, we use the normalized temperature-scaled cross entropy loss function^73^. In a given batch with *n* data points, each data point *x* is augmented twice to create $x_i$ and $x_j$. The positive pair is forward propagated through the CNN encoder to extract representations $h_i$ and $h_j$. These representation vectors are passed through the projection head to yield $z_i$ and $z_j$. This loss function is represented in Equation 2.

2 $$\begin{aligned} L_{i,j} = -\log \left( \frac{\exp \left( \frac{\text {sim}(\textbf{z}_i, \textbf{z}_j)}{\tau } \right) }{\sum _{k=1}^{2N} \textbf{1}_{[k \ne i]} \exp \left( \frac{\text {sim}(\textbf{z}_i, \textbf{z}_k)}{\tau } \right) } \right) \end{aligned}$$

Although there is no negative sample mining, every sample in the training batch, aside from the positive pair, is considered a negative sample. The dot product between two data points gives the cosine similarity, sim($z_i$, $z_j$). τ is the temperature parameter. The loss function aims to update the model weights such that the model maximizes the cosine similarity between the representations of the positive pair while minimizing the similarity between the positive and negative samples.

The training procedure is as recommended by SimCLR^39^. We selected hyper-parameters of learning rate, temperature, learning-rate scheduler, latent-dimension size, projection-dimension size, batch size, weight decay, and training epochs using random search. The final model was trained using the LambdaLR learning rate scheduler, with a starting learning rate of 0.001 and a linear warm up period of 10 epochs to reach a maximum learning rate of 0.01. Temperature was set to 0.13. The representation dimension, h, was a 128-dimensional vector while the projection dimension was 64. Batch size was set to 1024. Weight decay was set to 0.0001. The model was trained for 50 epochs on one RTX4090 GPU on Dataset C.

The choice and configuration of image augmentations play a critical role in contrastive learning. In our approach, we apply a series of random transformations to each image in a pair, independently and in a fixed order, with each augmentation having its own probability p of being applied. First, we apply a color jitter on all channels (brightness=0.4, contrast=0.4, saturation=0.4, hue=0.2, p=0.5). We then apply a random rotation between -180$^\circ$ and 180$^\circ$ (p=0.5), followed by random horizontal and vertical flips (both p=0.5). Next, an affine translation of up to 15 pixels off-center in both the x and y directions is added (p=0.5), after which we apply a Gaussian blur (p=0.5) using a 3-pixel kernel and a sigma chosen randomly between 0.5 and 3.0. Finally, we randomly crop the image to between 50% and 100% of its original size, then resize it back to 75 $\times$ 75 (p=0.5).

These parameters were selected through careful visual evaluation of their effects on immunofluorescent images, ensuring that we preserved crucial fluorescent signals while encouraging the model to generalize across a wide range of cellular phenotypes.

### Cell phenotype classification model

A logistic regression model was trained on the 80% split training data of Dataset D from scikit-learn package (version 1.5.2). The model was trained for a maximum of 10,000 iterations until convergence. Accuracy, precision, recall, and F1-score were calculated for each class on the test split.

### Feature Robustness Analysis

For each cell present in our dataset, we generated features using our contrastive learning feature set and a set of engineered cell features on original and perturbed images. Perturbations were applied as Gaussian blur with variable standard deviations, intensity variations by scaling the raw intensities within a range of $\pm 20\%$, and isotropic resizing by $\pm 10\%$ followed by center cropping or padding to restore image original size. We assessed the pairwise cosine distance between features of the original and perturbed cell image pairs.

### Outlier detection analysis

We evaluated the performance of features extracted through contrastive learning in detecting true outliers and compared the performance to engineered features on Spiked-In WSIs (Dataset E). The contrastively learned features have 128 dimensions, whereas the engineered features have 368 dimensions (see engineered feature extraction). For three outlier detection algorithms (COPOD, ECOD, iForest), we evaluated the area under the ROC curve for the first 2,500 events (representing approximately 0.1% of the data in a WSI). All outlier detection algorithms were implemented using pyOD^48^. For all outlier detection algorithms, the level of contamination was set to 0.001. The number of estimators used in iForest was 100.

### Clustering analysis

We evaluated clustering performance using two unsupervised algorithms: K-means and Leiden community detection. For K-means, clustering was performed using the KMeans implementation from scikit-learn with the number of clusters set to 10 and a fixed random seed to ensure reproducibility. For Leiden clustering, a k-nearest neighbors (k-NN) graph was constructed using cosine similarity with neighborhood size set to 15. The resulting graph was symmetrized and passed to the Leiden algorithm (via the leidenalg package), using the modularity vertex partition scheme. Clustering results from both methods were evaluated against ground truth labels (Dataset D) using standard metrics (Adjusted Rand Index, Normalized Mutual Information, Homogeneity, Completeness). The ratio between WBCs and rare cells was iterated over from 0.5-10 to identify how clustering performance was impacted at different balancing ratios.

### Contrived cell phenotype identification model

Two of nine spiked-in slides (Dataset E), one containing SK-BR-3 cells only and another containing HPAEC cells only, were used to train a multiclass classifier to evaluate the ability of the embeddings to learn cell-type on a limited set of data. The multiclass classifier consisted of a two-layer fully connected multilayer perceptron model. The model was trained for 100 epochs with a learning rate of 0.01 and optimized with the Adam optimizer. The training data consisted of 6,636 events classified as others, 683 events classified as SK-BR-3, and 408 events classified as HPAEC. We evaluated the performance of the representation-learning model by applying the classifier across the 7 remaining independent slides. For each slide, we calculated the precision, recall, and F1-score. We aggregated all of the test slides and calculated overall performance metrics.

### Patient cell phenotype identification and classification model

Two of eight metastatic breast cancer slides (Dataset F) were used to train a multiclass classifier to evaluate the ability of the embeddings to learn cell-type on a limited set of data. The multiclass classifier consisted of a three-layer fully connected multilayer perceptron model. The model was trained for 50 epochs with a learning rate of 0.001, a weight decay of 0.0001 and optimized with the Adam optimizer. The training data consisted of 5,000 events classified as others, 177 events classified as CTCs, and 2734 events classified as imCTCs. We evaluated the performance of the representation-learning model by applying the classifier across the 6 remaining test slides. For each slide, we calculated the precision, recall, and F1-score, in addition to overall performance metrics.

## Data Availability

The datasets generated and used for training and fine-tuning the models have been deposited in the BloodPAC Data Commons and are available under accession ID BPDC000147.

## Declarations

### Code Availability

All the codes developed for the model training and analysis are available at https://github.com/CSI-Cancer/deep_phenotyping. The image annotation tool, AnnotateEZ, is available to the public at https://github.com/aminnaghdloo/annotateEZ.
